# Correlation between Extraocular Muscle Size Measured by Computed Tomography and the Vertical Angle of Deviation in Thyroid Eye Disease

**DOI:** 10.1371/journal.pone.0148167

**Published:** 2016-01-28

**Authors:** Ju-Yeun Lee, Kunho Bae, Kyung-Ah Park, In Jeong Lyu, Sei Yeul Oh

**Affiliations:** Department of Ophthalmology, Samsung Medical Center, Sungkyunkwan University School of Medicine, Seoul, Korea; University of Birmingham, UNITED KINGDOM

## Abstract

The aim of this study was to investigate extraocular muscle (EOM) volume and cross-sectional area using computed tomography (CT), and to determine the relationship between EOM size and the vertical angle of deviation in thyroid eye disease (TED). Twenty-nine TED patients (58 orbits) with vertical strabismus were enrolled in the study. All patients underwent complete ophthalmic examination including prism, alternate cover, and Krimsky tests. Orbital CT scans were also performed on each patient. Digital image analysis was used to quantify superior rectus (SR) and inferior rectus (IR) muscle cross-sectional areas and volumes. Measurements were compared with those of controls. The correlation between muscle size and degree of vertical angle deviation was evaluated. The mean vertical angle of deviation was 26.2 ± 4.1 prism diopters. The TED group had a greater maximum cross-sectional area and EOM volume in the SR and IR than the control group (all *p*<0.001). Area and volume of the IR were correlated with the angle of deviation, but the SR alone did not show a significant correlation. The maximum cross-sectional area and volume of [Right IR + Left SR − Right SR − Left IR] was strongly correlated with the vertical angle of deviation (*P*<0.001). Quantitative CT of the orbit with evaluation of the area and volume of EOMs may be helpful in anticipating and monitoring vertical strabismus in TED patients.

## Introduction

Thyroid eye disease (TED), also called Graves’ orbitopathy, is considered as an autoimmune disease characterized by lymphocytic infiltration of the retrobulbar tissues, including the extraocular muscles (EOMs). After the inflammatory phase, fibrosis of these tissues replaces inflammation. As a result, magnetic resonance imaging or computed tomography (CT) scans reveal EOM enlargement in nearly 70% of adult patients with TED [1]. Enlargement of the inferior rectus (IR) and medial rectus (MR) muscles, which are most often involved, result in vertical strabismus. [2].

Restricted EOM motility and strabismus in TED is a common condition resulting from fatty and fibrotic changes due to cell-mediated and humoral immune processes. Most patients undergo spontaneous remission of the active phase of TED within years. However, despite of the remission, the resultant fibrosis and fat expansion typically persist [3]. Previous studies have shown an association between muscle fibrosis and restriction in ocular motility [3, 4]. Regarding inconsistency in the angle of deviation, one of the most challenging aspects of thyroid disease is evaluating and monitoring restrictive strabismus.

Various imaging techniques have been used to evaluate morphological and structural changes of EOMs in TED. Since the introduction of CT for the examination of EOM enlargement in patients with TED [5, 6], it has become a useful technique to assess restriction in ocular motility. CT provides rapid and accurate visualization of orbital structures and muscle volume investigation with CT has proven useful as a tool in diagnosis, prognosis, and follow-up in TED patients [4, 7–9].

Several studies have shown an association between muscle enlargement on CT and clinical evidence of restriction in ocular motility [4, 10–13]. Hallin and associates reported that muscle area and total muscle volume were highly correlated with clinical signs including the limitation of ocular motility and optic nerve involvement [4]. Chen et al. established a significant relationship between the width of the IR muscle and limitation of supraduction in patients with TED [10]. Although restrictive strabismus triggered due to impaired ocular motility can easily be observed in TED, to the best of our knowledge, no consensus exists regarding the quantification of strabismus using CT-based determination of EOM size.

Therefore, the purpose of this study was to examine the significance of EOM enlargement as established by CT-based size determination and to evaluate the relationship between EOM size and angle of deviation in stable TED. To simplify the analysis, only the relationship between vertical strabismus and vertically-placed EOMs was established in this study.

## Methods

This study was a hospital-based retrospective observational study of patients who were examined at Samsung Medical Center by a single clinician (S.O.) between March 2011 and June 2013. This study was approved by the ethics committee of Samsung Medical Center Institutional Review Board, and followed the tenets of the Declaration of Helsinki.

We recruited all patients who were diagnosed with TED with vertical strabismus over 8 prism diopters (PD). All subjects underwent complete ophthalmic examination. The angle of deviation was measured in PD using the alternate prism and cover test (APCT) with fixation at 33 cm and 6 m. The Krimsky reflex method was also used in patients whose ocular motility was restricted more than -2 grades of limitation, as described by Feldon and colleagues [14]. We instructed each patient to focus on a target 33 cm away and applied a prism over the patient’s dominant eye with a light source. When the corneal light reflection was located at the center of each cornea, we measured the angle of deviation. Only patients who had undergone ophthalmologic examination within 1 month of CT imaging were included in the study. Patients were excluded if they had a history of strabismus surgery, cranial nerve palsy, inflammatory conditions such as pseudotumor and myositis, any orbital mass, or a systemic disease such as myasthenia gravis or chronic progressive external ophthalmoplegia. Subjects with contraindications to CT scanning were also excluded. Patient records were anonymized and de-identified prior to analysis.

The state of TED—active or stable—was recorded by the oculoplasty specialist at the time of clinical evaluation. Active TED was defined by the presence of a change in clinical examination since the prior exam, or by signs of inflammation including pain on eye movement, chemosis, or fluctuating eyelid edema/erythema. Stable TED was diagnosed when there was neither change in clinical examination nor findings of inflammation over a 6-month period. All patients were euthyroid and had been stable for longer than 6 months before surgery. To minimize the time course of changes in the muscles, only stable TED patients with a vertical angle of deviation were included in this study.

To establish normal EOM values, we included an age-matched control group of 10 normal, orthotropic volunteers with a mean age of 55.0 ± 10.7 years (range, 47 to 67 years), of which four were male and 6 female. Each control underwent ophthalmologic examination to verify normal visual acuity, ocular motility, and ocular anatomy. None had undergone any prior ocular surgery except for cataract surgery.

### Measurement methods

**Fig 1:**
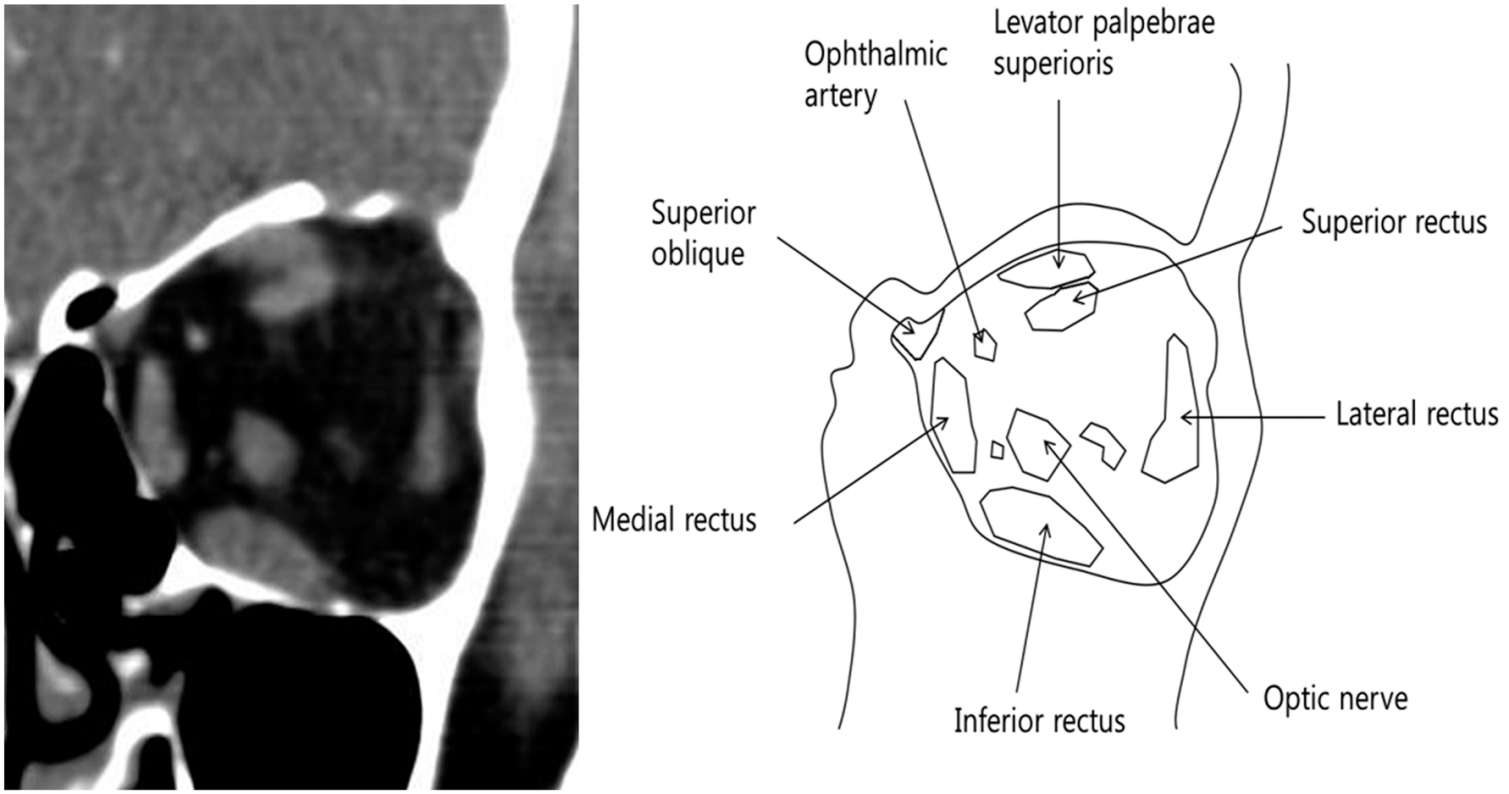
Contrast CT image and schematic drawing of the coronal plane orbit. (a) Contrast CT image through the mid-left orbit in a patient with mild TED. The superior rectus muscle (SR) can be distinguished from the superior ophthalmic vein and the levator palpebrae. (b) Schematic drawing of the coronal plane orbit. The area of the IR appears significantly larger than the area of the SR.

Sequences of CT slides were obtained in 3-mm-thick coronal, continuous sections using a multi slice spiral CT scanner (Mx8000 IDT; Philips, Eindhoven, The Netherlands). The 3-mm coronal CT slabs were reformatted from contiguous axial slices of the orbit, which includes the space between the interzygomatic line and the orbital apex. A soft tissue window (grayscale) setting was used to differentiate orbital muscle, fat, and bone tissue. Orbital soft tissue thresholds were set at 30 to 100 Hounsfield units (HU) for muscle tissue. A digital image CT viewer and Hypersnap^™^ 6 (Hyperionics Technology, Murrysville PA, USA) were used to capture the CT images (Fig 1). Image analysis was similar to published methods [15, 16]. Every digital CT image was converted to 8-bit tagged image file format (TIFF) with locally developed software. For accurate measurement, quantitative estimation of EOM size was performed manually with the Image J program (National Institutes of Health, Rockville Pike, Bethesda, MD, USA). An equal scaling index was included in every CT image, and a consistent method of measurement of EOM size was used to avoid possible measurement errors. We performed manual outlining of the superior rectus (SR) and inferior rectus (IR) at the primary gaze position with a tablet monitor, and area calculation was automatically performed in Image J. The maximum value of the vertical EOMs in each subject was used for analysis.

Two independent observers (J.L., K.B.), who were blinded to clinical strabismus measurements, obtained every measurement twice to ensure data consistency, and the mean of the two measurements was recorded. Inter-observer reproducibility was evaluated based on the intraclass correlation coefficient (ICC). The intra-observer ICC value was also calculated after analysis.

### Area and volume calculation methods

**Fig 2:**
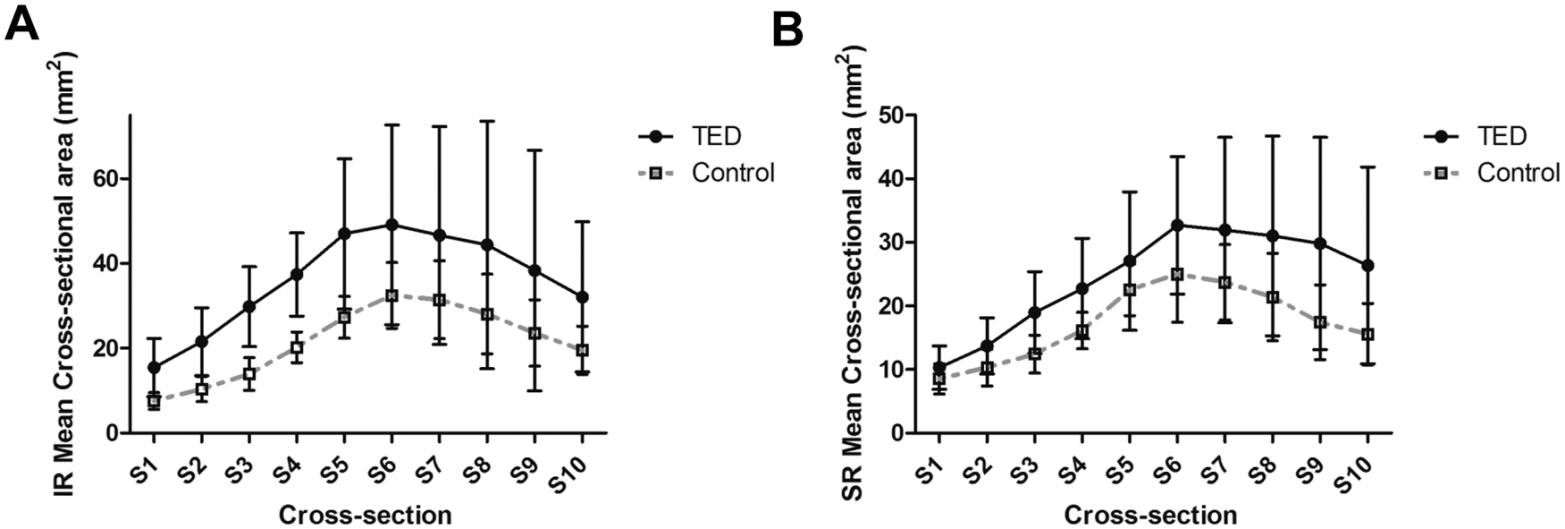
**(A)** Serial mean cross-sectional areas of the inferior rectus muscle (mm^2^). **(B)** Serial mean cross-sectional areas of the superior rectus muscle (mm^2^).

EOM area was measured anteriorly and posteriorly from the maximum cross-sectional area (MCA). Among the sequences of CT image slides, the section with the maximum value was selected as the MCA using the calculated values of SR and IR area. Five anterior and four posterior sections from the MCA were used to estimate the EOM volume (Fig 2. S1: anterior section—S10: posterior section). We assumed linear interpolation of the data over the slice gap. Therefore, the volume, *V*, for each muscle was estimated using the formula

$$V=\;S_{G}\overset{N-1}{\underset{n=1}{\sum^{\;}}}\frac{S_{n}+S_{n+1}}{2}$$

where S_G_ is the slice gap, N is the total number of slices upon which areas were measured for that muscle, and S_n_ is the muscle area of slice n.

### Data analysis

We evaluated trends in vertical strabismus versus various combinations of bilateral SR and IR muscle values. To standardize vertical strabismus, the angle of deviation was defined relative to the right eye. If the right eye was higher, then a positive angle was assigned; if the right eye was lower, then a negative angle was assigned.

We evaluated correlations between the vertical angle of deviation and combinations of EOM area and volume. Variation was determined as varied combination considering the reciprocal action of the EOM on vertical strabismus. Thus, the right SR (RSR), right IR (RIR), left SR (LSR), left IR (LIR), [RSR + LIR], [RIR + LSR], [RSR—RIR], [LSR—LIR], and [(RSR+LIR)-(RIR+LSR)] were all evaluated. We used both the maximum cross-sectional area and the EOM volume for analysis. We determined the mean area and the standard deviation for each muscle. The normative average from the control group was compared with the average muscle areas in our study cohort using paired t tests. We deduced correlations using the linear regression analysis method with Bonferroni correction. All statistical analyses in this study were done using SPSS 18 for Windows (SPSS, Chicago, IL, USA). Inter-observer variability and intra-observer repeatability were analyzed using the intraclass correlation coefficient (ICC). A *p* value less than 0.05 was considered significant.

## Results

We enrolled 29 TED patients including 14 females and 15 males in this study. The mean age was 54.1 ± 11.4 years (range, 27 to 74 years). Total duration of TED was 56.93±45.25 months (range, 18 to 180 months). The mean vertical angle of deviation was 26.2 ± 14.1 PD (range, 8 to 65 PD).16 patients (55.2%) were treated with steroid, and 6 patients (20.7%) were treated with radiation therapy. No patient had surgical decompression.

Average values and ranges for the areas of the SR and IR in TED patients and normal control patients are shown in Table 1. The mean maximum area and EOM volume of the IR were significantly larger than the SR in both the TED group and the control group (*P*<0.05). The TED group had a significantly larger maximum EOM area than the control group; mean values of maximum EOM area were 37.5 ± 15.7 mm^2^ for the SR and 56.9 ± 26.2 mm^2^ for the IR in TED patients. Compared with the control group, these average areas exceeded the upper limit of the normal range. Mean volume estimates were 681.9 ± 244.9 mm^3^ for the SR and 949.5 ± 349.6 mm^3^ for the IR in TED patients (Table 2). The EOM volume was greater than the control group for both the SR and IR, and was more significant.

**Table 1:** Average maximal muscle areas (mm^2^) of thyroid eye disease (TED) patients versus normal control patients.

Extraocular muscle	TED	Control group	*P*-value
RSR	36.4 (17.6–80.0)	27.0 (20.6–36.2)	0.04
RIR	56.3 (26.3–147.4)	35.1 (25.3–47.8)	<0.01
LSR	38.9 (20.1–79.6)	27.5 (21.8–36.6)	0.03
LIR	57.4 (22.0–130.5)	35.7 (26.1–44.1)	<0.01

RSR, right superior rectus; RIR, right inferior rectus; LSR, left superior rectus; LIR, left inferior rectus.

**Table 2:** Average muscle volume (mm^3^) of thyroid eye disease (TED) patients versus normal control patients.

Extraocular muscle	TED	Control group	*P*-value
RSR	669.9 (360.7–1387.6)	471.7 (356.7–586.6)	<0.01
RIR	919.8 (490.2–1972.8)	601.7 (504.2–739.9)	<0.01
LSR	693.9 (376.5–1446.0)	494.0 (397.9–661.2)	<0.01
LIR	979.2 (455.3–1863.7)	604.4 (517.0–703.7)	<0.01

RSR, right superior rectus; RIR, right inferior rectus; LSR, left superior rectus; LIR, left inferior rectus.

Negative hypertropia (NHT) was observed in 17 patients and positive hypertropia (PHT) was observed in 12 patients. The mean angle of deviation was 26.4 PD in PHT (range, 15 to 50 PD), and 26.0 PD in NHT (range, 8 to 65 PD). There was a significant correlation between vertical strabismus and the value of RIR, LIR, [RIR + LSR], [RSR—RIR], [LSR—LIR] and [(RSR+LIR)-(RIR+LSR)] in both maximum area and volume (Table 3).

**Table 3:** Correlation between vertical strabismus and extraocular muscle size (maximum cross section area, volume) of thyroid eye disease patients.

	Maximum cross section area (mm^2^)		Volume (mm^3^)	
Variable	R-square	*p* value	R-square	*p* value
RSR	0.03	1.00	0.05	1.00
LSR	0.05	1.00	0.02	1.00
RIR	0.46	**<0.01**	0.37	**<0.01**
LIR	0.27	**0.04**	0.25	0.05
RSR+LIR	0.20	0.21	0.20	0.21
RIR+LSR	0.44	**<0.01**	0.32	**0.02**
RSR—RIR	0.42	**<0.01**	0.41	**<0.01**
LSR—LIR	0.37	**<0.01**	0.45	**<0.01**
(RSR+LIR)-(RIR+LSR)	0.66	**<0.01**	0.65	**<0.01**

RSR, right superior rectus; RIR, right inferior rectus; LSR, left superior rectus; LIR, left inferior rectus.

The maximum cross-sectional area of [(RSR+LIR)-(RIR+LSR)], which had the most significant correlation with vertical strabismus, has a regression equation that can be derived as follows:

Vertical angle of deviation (PD) = 0.62×[(RSR+LIR)−(RIR+LSR)] − 3.05

Therefore, for each 1 mm^2^ increase in [(RSR+LIR)-(RIR+LSR)], the vertical angle of deviation increases by 0.62 PD, with an intercept of -3.05.

**Fig 3:**
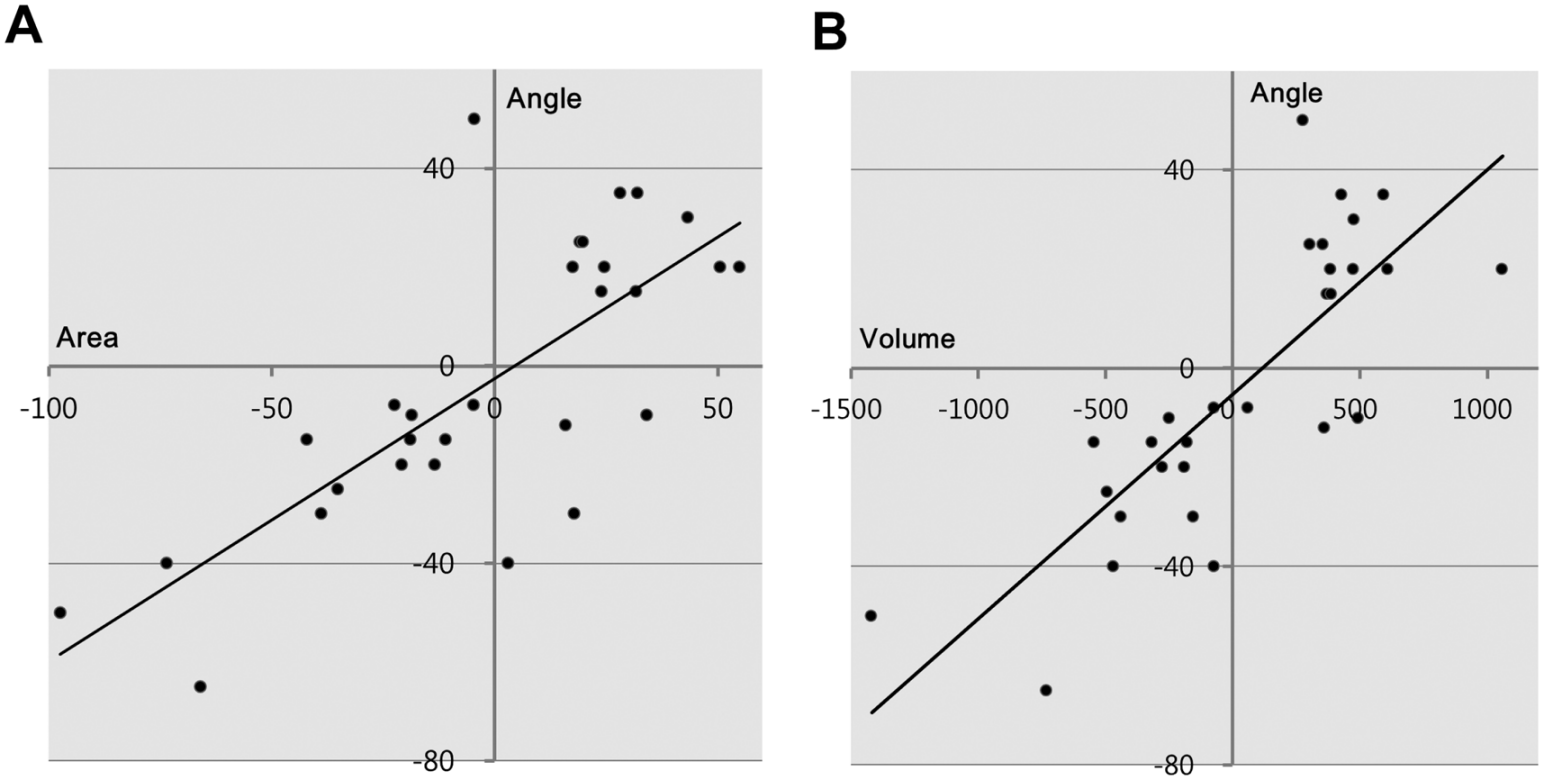
Trend lines between EOM and the vertical angle of deviation for [(RSR+LIR)-(RIR+LSR)] (A, maximum cross-sectional area; B, volume).

The *P*-value of the linear regression analysis was <0.001, which indicated strong predictive power. The maximum cross sectional area and volume of [(RSR+LIR)-(RIR+LSR)] were graphed with trend lines (Fig 3).

The intra-examiner reproducibility values were 0.824 and inter-examiner reproducibility values were 0.881. The intra- and inter-examiner ICC reproducibility for the area and the volume calculation of muscles all exceeded 0.75 (excellent).

## Discussion

Strabismus in TED patients is distinct from idiopathic strabismus, and is characterized as restrictive strabismus due to EOM fibrosis. In TED, reversible inflammation can result in decreased EOM size and improvements in the angle of deviation, but these improvements often do not return to normal [3]. In the stable phase, atrophy and fibrosis of muscle fibers are evident, and fibrous strands extend into adjacent adipose tissues [17]. Fibrosis in stable TED often leads to greater restriction than in the active phase, where fibrosis is incomplete [3]. This finding suggests that the cause of the angle of vertical deviation may be the fibrotic enlargement of EOMs.

Angle of deviation can vary with head position, especially when persistent EOM fibrosis has developed in the chronic phase of TED. Because restrictive strabismus results from fibrotic EOM enlargement, measuring EOM volume may complement strabismus evaluation and follow-up care of TED patients. Prior studies revealed the relationship between muscle enlargement and restriction in ocular motility [3, 4], but no reports have investigated the correlation between muscle enlargement and the angle of deviation.

To clarify the relationship between fibrotic EOM enlargement and the angle of vertical deviation, CT images were used to evaluate EOM size in patients with TED. Even with improved imaging techniques, the area of the SR muscle is difficult to measure because the levator palpebrae and the superior ophthalmic vein are in such a close relationship with SR muscle that they are difficult to differentiate. This difficulty is amplified in TED patients when the EOMs are enlarged [18]. Most researchers measure these structures together as the SR group, which likely results in a slightly larger muscle volume [18–25]. Others choose to exclude these muscles [26]. In this study, we tried to exclude the levator palpebrae muscle and the superior ophthalmic vein in measurements of the area and volume of the SR, as they can affect the results. We therefore excluded 2 of 29 CT scans (6.9%) of poor quality in which it was difficult to separate these structures for accurate measurement.

In the literature, the IR muscle, the MR muscle, and the SR muscle group are the most commonly enlarged muscles in TED patients. However, the LR muscle had an average diameter within the normal range [4]. Since the LR along with the MR are the primary EOMs that direct horizontal eye movement, we exclusively focused on vertical EOMs to rule out the effect of LR, which is the least affected muscle in TED. Considering the reciprocal action of the SR and IR on both eyes, we tried to evaluate the effect of extensive cases of vertical EOMs on vertical strabismus. Therefore, we analyzed not only each vertical EOMs but also various additive and subtractive formulas including [RSR + LIR], [RIR + LSR], [RSR—RIR], [LSR—LIR], and [(RSR+LIR)-(RIR+LSR)] to ascertain the relationship between vertical strabismus and vertically-placed EOMs.

This study revealed a strongly positive relationship between quantitative CT measurement of EOM size and angle of vertical deviation in TED. Among single EOMs, the maximum cross-sectional area and volume of the IR were correlated with the angle of vertical deviation, but the SR alone did not show a significant correlation. These results accord well with previous studies. Dagi et al. reported a trend toward increased motility restriction with increased EOM diameter in TED [3]. In their study, the mean diameter and ocular motility restriction were greater in IR than SR. Because angle of deviation is affected by ocular motility restriction, the IR muscle is likely to affect the angle of vertical deviation in TED.

It is notable that the maximum cross-sectional area and volume of [(RSR+LIR)-(RIR+LSR)] was most correlated with the angle of vertical deviation in this study. Considering the reciprocal action of vertical EOMs on vertical strabismus, we analyzed multiple variations in TED. Variation considering the reciprocal action for four different vertical EOMs in both eyes showed the most significant relationship with restrictive strabismus. We found that patients whose IR muscle combination size (RIR+LIR, or RIR-LIR) was similar showed different amounts of vertical deviation depending on SR muscle size. Although SR muscle size did not separately affect the angle of vertical deviation, the size of the IR and SR muscle combination considering reciprocal action is more reliable than single EOM size for assessing the angle of vertical deviation in TED.

Volume measurement is known to be more directly indicative of EOM enlargement than either muscle diameter or cross-sectional area estimation [22]. However, several quantitative measures other than total EOM volume are highly correlated with EOM enlargement [9]. In our study, the volume of EOM showed a strong correlation with the angle of deviation, and maximum cross-sectional area showed similar results of correlation. This result implies that maximum cross-sectional EOM area, rather than EOM volume, can be used to monitor progression in Graves’ orbitopathy.

This study has shown that quantitative CT evaluation of the extraocular muscles is one of the most important parameters in assessing vertical strabismus as well as ocular motility restriction in patients with TED. If EOM measurement is used to monitor progression of Graves’ orbitopathy, changes in muscle size should be studied. In addition, some form of image registration is necessary to identify equivalent section of the muscle.

The following limitations were encountered in this study. First, we used a single center for this study and all subjects had the same ethnicity. Some of the results may thus not be valid for other ethnic groups. Second, our data are taken from a single time point. While it is true that we found a strong correlation between EOM area and/or volume and the magnitude of strabismus in stable TED, we cannot conclude that changes in EOM size over time in a particular patient will be correlated with changes in the angle of strabismus in the active phase. A longitudinal study is required to further clarify this relationship. This raises another concern, which is the benefit of serial CT versus the risk associated with repeat radiation exposure. TED patients with strabismus do tend, on average, to be older than TED patients as a whole, but current recommendations are still to limit radiation exposure in all patients whenever possible.

In conclusion, both the cross-sectional area and EOM volume were greater than the norm in the muscles, and there was a strong correlation between these parameters and the vertical angle of deviation. Maximum cross-sectional area rather than total EOM volume can be used to monitor Graves’ orbitopathy. Quantitative CT imaging of the orbit with evaluation of the area and volume of EOMs may be helpful in anticipating and monitoring strabismus in TED patients.

## Supporting Information

S1 Table Maximum plane area and volume of extraocular muscle measured by computed tomography. (DOCX)
