# Supplementary material for: Correlation between Extraocular Muscle Size Measured by Computed Tomography and the Vertical Angle of Deviation in Thyroid Eye Disease
Source: PLoS One. 2016 Jan 28;11(1):e0148167. doi: 10.1371/journal.pone.0148167 (PMC4731061; doi:10.1371/journal.pone.0148167)
Supplement: S1 Table — (DOCX) [file pone.0148167.s001.docx]

| **patient** | **age** | **vertical deviation** | | **Maximum plane area of extraocular muscle** | | | | | | | | |
| --- | --- | --- | --- | --- | --- | --- | --- | --- | --- | --- | --- | --- |
|  |  |  |  | **RIR** | **RSR** | **LIR** | **LSR** | **RSR+LIR** | **LSR+RIR** | **RSR-RIR** | **LSR-LIR** | **RSR+LIR-RIR-LSR** |
| 1 | 53 | -15.0 | LHT | 57.7 | 26.9 | 42.4 | 22.5 | 69.3 | 80.2 | -30.8 | -19.9 | -10.9 |
| 2 | 48 | -15.0 | LHT | 43.6 | 29.8 | 35.9 | 40.9 | 65.7 | 84.5 | -13.8 | 5.0 | -18.8 |
| 3 | 49 | -8.0 | LHT | 147.4 | 74.2 | 130.5 | 79.6 | 204.7 | 227.0 | -73.2 | -50.9 | -22.3 |
| 4 | 52 | 50.0 | RHT | 49.9 | 24.2 | 54.6 | 33.4 | 78.8 | 83.3 | -25.7 | -21.1 | -4.5 |
| 5 | 39 | -30.0 | LHT | 41.2 | 25.6 | 53.6 | 20.1 | 79.2 | 61.3 | -15.6 | -33.5 | 17.9 |
| 6 | 39 | -50.0 | LHT | 130.8 | 17.6 | 39.6 | 23.7 | 57.2 | 154.5 | -113.2 | -15.9 | -97.3 |
| 7 | 57 | 15.0 | RHT | 30.6 | 32.7 | 65.8 | 36.1 | 98.5 | 66.7 | 2.1 | -29.7 | 31.8 |
| 8 | 43 | -10.0 | LHT | 66.8 | 80.0 | 83.3 | 62.4 | 163.3 | 129.2 | 13.1 | -21.0 | 34.1 |
| 9 | 52 | -40.0 | LHT | 74.6 | 28.5 | 29.4 | 56.7 | 58.0 | 131.3 | -46.1 | 27.3 | -73.4 |
| 10 | 73 | 20.0 | RHT | 43.4 | 30.5 | 92.0 | 24.1 | 122.5 | 67.5 | -12.9 | -67.9 | 55.0 |
| 11 | 36 | -65.0 | LHT | 72.4 | 31.6 | 42.5 | 67.5 | 74.1 | 140.0 | -40.8 | 25.0 | -65.8 |
| 12 | 41 | -30.0 | LHT | 65.8 | 25.1 | 22.0 | 20.2 | 47.1 | 86.0 | -40.7 | -1.8 | -38.9 |
| 13 | 74 | -40.0 | LHT | 84.7 | 44.0 | 79.0 | 35.2 | 123.0 | 119.9 | -40.7 | -43.8 | 3.1 |
| 14 | 69 | 15.0 | RHT | 62.7 | 74.5 | 77.9 | 65.6 | 152.4 | 128.4 | 11.8 | -12.3 | 24.1 |
| 15 | 52 | 25.0 | RHT | 50.7 | 32.7 | 77.6 | 40.5 | 110.3 | 91.2 | -18.0 | -37.1 | 19.2 |
| 16 | 77 | 20.0 | RHT | 26.5 | 25.7 | 53.7 | 28.1 | 79.3 | 54.7 | -0.8 | -25.5 | 24.7 |
| 17 | 66 | 30.0 | RHT | 41.5 | 27.8 | 79.9 | 22.9 | 107.7 | 64.4 | -13.7 | -57.1 | 43.3 |
| 18 | 50 | -12.0 | LHT | 36.6 | 44.7 | 48.9 | 41.1 | 93.6 | 77.7 | 8.1 | -7.8 | 15.9 |
| 19 | 54 | 35.0 | RHT | 42.3 | 38.1 | 67.8 | 35.5 | 105.9 | 77.7 | -4.1 | -32.3 | 28.2 |
| 20 | 66 | -20.0 | LHT | 61.2 | 33.6 | 41.6 | 34.7 | 75.1 | 95.9 | -27.6 | -6.9 | -20.8 |
| 21 | 52 | -8.0 | LHT | 48.5 | 33.5 | 36.5 | 26.2 | 70.1 | 74.8 | -15.0 | -10.3 | -4.7 |
| 22 | 72 | -20.0 | LHT | 36.9 | 30.3 | 22.4 | 29.3 | 52.8 | 66.2 | -6.5 | 6.9 | -13.4 |
| 23 | 55 | -15.0 | LHT | 65.8 | 21.7 | 32.0 | 29.9 | 53.7 | 95.7 | -44.1 | -2.1 | -42.0 |
| 24 | 40 | 25.0 | RHT | 40.3 | 40.4 | 47.4 | 27.6 | 87.8 | 67.9 | 0.1 | -19.8 | 19.9 |
| 25 | 51 | 20.0 | RHT | 37.5 | 33.3 | 97.2 | 42.5 | 130.5 | 80.0 | -4.2 | -54.8 | 50.6 |
| 26 | 49 | 35.0 | RHT | 26.3 | 50.9 | 66.5 | 59.0 | 117.4 | 85.3 | 24.6 | -7.5 | 32.1 |
| 27 | 44 | -25.0 | LHT | 63.5 | 38.1 | 43.9 | 53.5 | 82.0 | 117.1 | -25.5 | 9.6 | -35.1 |
| 28 | 59 | -10.0 | LHT | 39.4 | 26.6 | 28.6 | 34.3 | 55.2 | 73.7 | -12.8 | 5.7 | -18.5 |
| 29 | 57 | 20.0 | RHT | 44.0 | 22.7 | 72.7 | 33.8 | 95.4 | 77.8 | -21.4 | -39.0 | 17.6 |

| **patient** | **age** | **vertical deviation** | | **Volume of extraocular muscle** | | | | | | | | |
| --- | --- | --- | --- | --- | --- | --- | --- | --- | --- | --- | --- | --- |
|  |  |  |  | **RIR** | **RSR** | **LIR** | **LSR** | **RSR+LIR** | **LSR+RIR** | **RSR-RIR** | **LSR-LIR** | **RSR+LIR-RIR-LSR** |
| 1 | 53 | -15.0 | LHT | 967.3 | 521.1 | 588.7 | 459.9 | 1109.8 | 1427.2 | -446.2 | -128.8 | -317.4 |
| 2 | 48 | -15.0 | LHT | 771.8 | 618.2 | 695.4 | 720.5 | 1313.6 | 1492.3 | -153.7 | 25.1 | -178.8 |
| 3 | 49 | -8.0 | LHT | 1745.2 | 1387.6 | 1863.7 | 1446.0 | 3251.3 | 3191.2 | -357.6 | -417.7 | 60.1 |
| 4 | 52 | 50.0 | RHT | 732.4 | 475.4 | 922.5 | 388.2 | 1398.0 | 1120.6 | -257.0 | -534.3 | 277.4 |
| 5 | 39 | -30.0 | LHT | 717.1 | 425.8 | 736.6 | 599.8 | 1162.4 | 1316.9 | -291.2 | -136.8 | -154.5 |
| 6 | 39 | -50.0 | LHT | 1972.8 | 360.7 | 627.4 | 433.9 | 988.1 | 2406.7 | -1612.2 | -193.5 | -1418.6 |
| 7 | 57 | 15.0 | RHT | 676.3 | 578.3 | 1144.1 | 675.4 | 1722.3 | 1351.7 | -98.1 | -468.6 | 370.6 |
| 8 | 43 | -10.0 | LHT | 1159.8 | 1328.3 | 1344.4 | 1021.1 | 2672.7 | 2180.9 | 168.5 | -323.3 | 491.8 |
| 9 | 52 | -40.0 | LHT | 829.4 | 443.1 | 506.1 | 587.6 | 949.1 | 1417.1 | -386.3 | 81.6 | -467.9 |
| 10 | 73 | 20.0 | RHT | 745.6 | 597.9 | 1229.1 | 472.9 | 1827.1 | 1218.5 | -147.6 | -756.2 | 608.6 |
| 11 | 36 | -65.0 | LHT | 1228.6 | 550.2 | 1035.9 | 1088.1 | 1586.1 | 2316.7 | -678.4 | 52.2 | -730.6 |
| 12 | 41 | -30.0 | LHT | 1002.2 | 485.3 | 455.3 | 376.5 | 940.6 | 1378.7 | -516.9 | -78.8 | -438.1 |
| 13 | 74 | -40.0 | LHT | 1303.1 | 768.6 | 1184.9 | 722.1 | 1953.4 | 2025.3 | -534.6 | -462.7 | -71.8 |
| 14 | 69 | 15.0 | RHT | 1184.2 | 1216.3 | 1344.5 | 990.3 | 2560.8 | 2174.5 | 32.0 | -354.3 | 386.3 |
| 15 | 52 | 25.0 | RHT | 835.2 | 652.0 | 1246.8 | 760.9 | 1898.8 | 1596.1 | -183.3 | -485.9 | 302.7 |
| 16 | 77 | 20.0 | RHT | 501.7 | 504.5 | 1044.9 | 574.5 | 1549.4 | 1076.2 | 2.9 | -470.4 | 473.3 |
| 17 | 66 | 30.0 | RHT | 718.9 | 598.5 | 1139.0 | 542.4 | 1737.5 | 1261.3 | -120.4 | -596.6 | 476.2 |
| 18 | 50 | -12.0 | LHT | 810.6 | 885.0 | 1055.1 | 769.8 | 1940.2 | 1580.4 | 74.4 | -285.4 | 359.7 |
| 19 | 54 | 35.0 | RHT | 758.4 | 681.8 | 1154.9 | 651.4 | 1836.6 | 1409.8 | -76.6 | -503.4 | 426.8 |
| 20 | 66 | -20.0 | LHT | 901.8 | 638.9 | 719.3 | 733.9 | 1358.2 | 1635.7 | -263.0 | 14.5 | -277.5 |
| 21 | 52 | -8.0 | LHT | 849.9 | 667.8 | 717.9 | 607.7 | 1385.7 | 1457.6 | -182.1 | -110.2 | -71.9 |
| 22 | 72 | -20.0 | LHT | 681.4 | 628.8 | 474.0 | 611.1 | 1102.8 | 1292.5 | -52.6 | 137.1 | -189.7 |
| 23 | 55 | -15.0 | LHT | 1136.9 | 472.5 | 649.1 | 528.4 | 1121.6 | 1665.3 | -664.4 | -120.7 | -543.7 |
| 24 | 40 | 25.0 | RHT | 792.6 | 796.3 | 901.4 | 550.1 | 1697.6 | 1342.8 | 3.6 | -351.2 | 354.8 |
| 25 | 51 | 20.0 | RHT | 613.3 | 708.2 | 1825.2 | 860.8 | 2533.4 | 1474.0 | 94.9 | -964.5 | 1059.4 |
| 26 | 49 | 35.0 | RHT | 490.2 | 845.4 | 1136.9 | 899.2 | 1982.3 | 1389.4 | 355.2 | -237.7 | 592.9 |
| 27 | 44 | -25.0 | LHT | 1084.4 | 683.3 | 802.1 | 894.8 | 1485.4 | 1979.2 | -401.1 | 92.7 | -493.8 |
| 28 | 59 | -10.0 | LHT | 700.7 | 494.2 | 563.6 | 606.6 | 1057.8 | 1307.3 | -206.6 | 43.0 | -249.5 |
| 29 | 57 | 20.0 | RHT | 763.1 | 412.1 | 1287.0 | 550.5 | 1699.1 | 1313.6 | -351.0 | -736.5 | 385.5 |
